# Corrigendum to “Behavioral and brain signatures of substance use vulnerability in childhood” [Developmental Cognitive Neuroscience 46 (December) (2020) 100878]

**DOI:** 10.1016/j.dcn.2020.100891

**Published:** 2020-12-09

**Authors:** Kristina M. Rapuano, Monica D. Rosenberg, Maria T. Maza, Nicholas J. Dennis, Mila Dorji, Abigail S. Greene, Corey Horien, Dustin Scheinost, R. Todd Constable, B.J. Casey

**Affiliations:** a Department of Psychology, Yale University, New Haven, CT, United States; b Department of Psychology, University of Chicago, Chicago, IL, United States; c Interdepartmental Neuroscience Program, Yale School of Medicine, New Haven, United States; d Department of Radiology and Biomedical Imaging, Yale School of Medicine, New Haven, CT, United States

The authors regret that Figure 2 appeared incorrectly in the original article and should have been as follows:

**Figure:**
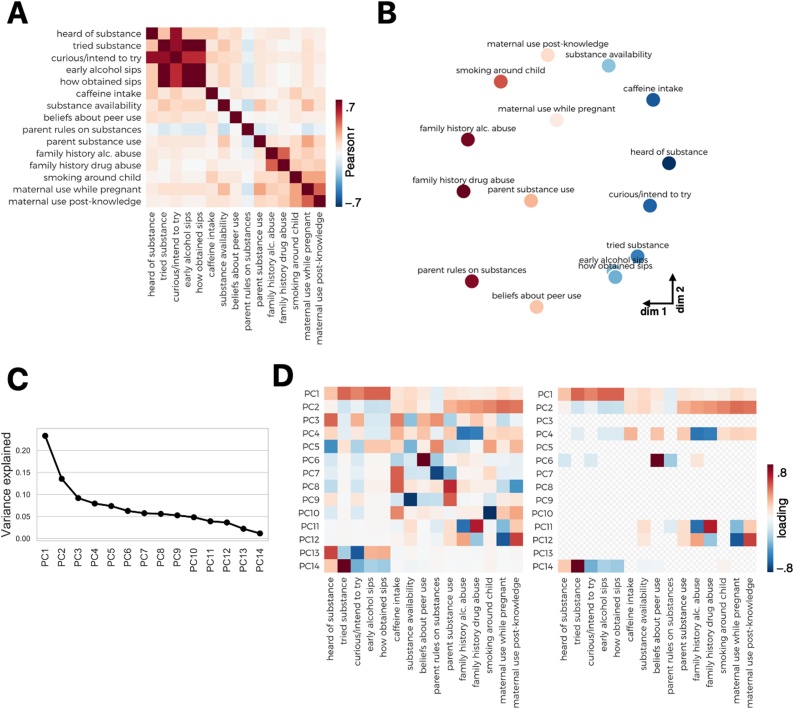

The authors would like to apologise for any inconvenience caused.

